# Guideline-Based Follow-Up Outcomes in Patients With Gastrointestinal Stromal Tumor With Low Risk of Recurrence: A Report From the Italian Sarcoma Group

**DOI:** 10.1001/jamanetworkopen.2023.41522

**Published:** 2023-11-06

**Authors:** Lorenzo D’Ambrosio, Elena Fumagalli, Tommaso Martino De Pas, Margherita Nannini, Alexia Bertuzzi, Silvia Carpano, Antonella Boglione, Angela Buonadonna, Danila Comandini, Silvia Gasperoni, Bruno Vincenzi, Antonella Brunello, Giuseppe Badalamenti, Elena Maccaroni, Giacomo Giulio Baldi, Alessandra Merlini, Andrea Mogavero, Francesca Ligorio, Elisabetta Pennacchioli, Fabio Conforti, Giulia Manessi, Sandra Aliberti, Francesco Tolomeo, Marco Fiore, Marta Sbaraglia, Angelo Paolo Dei Tos, Silvia Stacchiotti, Maria Abbondanza Pantaleo, Alessandro Gronchi, Giovanni Grignani

**Affiliations:** 1 Department of Medical Oncology, University of Turin, Turin, Italy; 2 San Luigi Gonzaga University Hospital, Orbassano, Italy; 3 Medical Oncology Unit 2, Medical Oncology Department, Fondazione IRCCS Istituto Nazionale dei Tumori di Milano, Milan, Italy; 4 Medical Oncology Division, Cliniche Humanitas Gavazzeni, Bergamo, Italy; 5 Previously at Unit of Sarcomas and Thymomas, European Institute of Oncology, Milan, Italy; 6 Oncology Unit. Department of Medical and Surgical Sciences, University of Bologna, 40138, Bologna, Italy; 7 Medical Oncology, Humanitas Cancer Center, Rozzano (MI), Italy; 8 Division of Medical Oncology 2, IRCCS Regina Elena National Cancer Institute, Rome, Italy; 9 Oncology Department, ASL Città di Torino, Turin, Italy; 10 Sarcoma and gastrointestinal tumors Unit, Centro di Riferimento Oncologico, Aviano, Italy; 11 Medical Oncology 1, Ospedale Policlinico San Martino, University of Genova, Genova, Italy; 12 Clinical Oncology Unit, Oncology Department and Robotic Surgery, AOU Careggi, Florence, Italy; 13 Medical Oncology, Università Campus Bio-Medico, Rome, Italy; 14 Medical Oncology 1, Istituto Oncologico Veneto IOV–IRCCS, Padua, Italy; 15 Medical Oncology, Department of Surgical, Oncological and Oral Sciences, University of Palermo, Palermo, Italy; 16 Department of Oncology, Azienda Ospedaliero-Universitaria delle Marche, 60126 Ancona, Italy; 17 Medical Oncology, Nuovo Ospedale Santo Stefano, Prato, Italy; 18 Sarcoma Unit, Candiolo Cancer Institute, FPO-IRCCS, Candiolo (TO), Italy; 19 Surgical Department, Melanoma and Sarcoma, European Institute of Oncology, Milan, Italy; 20 Sarcoma Service, Surgical Department, Fondazione IRCCS Istituto Nazionale dei Tumori di Milano, Milan, Italy; 21 Department of Medicine, University of Padua School of Medicine, Padua, Italy; 22 Medical Oncology 2, AOU Città della Salute e della Scienza di Torino, Turin, Italy

## Abstract

**Importance:**

Gastrointestinal stromal tumor (GIST) follow-up is recommended by international guidelines, but data on the role of follow-up in patients with low relapse risk are missing. For these patients, the potential benefit of anticipating recurrence detection should be weighed against psychological burden and radiologic examination loads in terms of costs and radiation exposure.

**Objective:**

To evaluate the outcomes of guideline-based follow-up in low-risk GIST.

**Design, Setting, and Participants:**

This multi-institutional retrospective cohort study involving Italian Sarcoma Group reference institutions evaluated patients with GIST who underwent surgery between January 2001 and June 2019. Median follow-up time was 69.2 months. Data analysis was performed from December 15, 2022, to March 20, 2023. Patients with GIST at low risk according to Armed Forces Institute of Pathology criteria were included provided adequate clinical information was available: primary site, size, mitotic index, surgical margins, and 2 or more years of follow-up.

**Exposures:**

All patients underwent follow-up according to European Society for Medical Oncology (ESMO) guidelines.

**Main Outcomes and Measures:**

The primary outcome was the number of tests needed to identify a relapse according to ESMO guidelines follow-up plan. Secondary outcomes included relapse rate, relapse timing, disease-free survival (DFS), overall survival (OS), GIST-specific survival (GIST-SS), postrelapse OS, secondary tumor rates, and theoretical ionizing radiation exposure. An exploratory end point, new follow-up schedule proposal for patients with low-risk GIST according to the observed results, was also assessed.

**Results:**

A total of 737 patients (377 men [51.2%]; median age at diagnosis, 63 [range, 18-86] years) with low-risk GIST were included. Estimated 5-year survival rates were 95.5% for DFS, 99.8% for GIST-SS, and 96.1% for OS. Estimated 10-year survival rates were 93.4% for DFS, 98.1% for GIST-SS, and 91.0% for OS. Forty-two patients (5.7%) experienced disease relapse during follow-up (9 local, 31 distant, 2 both), of which 9 were detected after 10 or more years. This translated into approximately 1 relapse detected for every 170 computed tomography scans performed, with a median radiation exposure of 80 (IQR, 32-112) mSv per patient. Nongastric primary tumor (hazard ratio [HR], 2.09; 95% CI, 1.14-3.83; *P* = .02), and *KIT* mutation (HR, 2.77; 95% CI, 1.05-7.27; *P* = .04) were associated with a higher risk of relapse. Second tumors affected 187 of 737 patients (25%), of which 56 were detected during follow-up and represented the primary cause of death in these patients.

**Conclusions and Relevance:**

In this cohort study on patients affected by low-risk GISTs, the risk of relapse was low despite a follow-up across 10 or more years. These data suggest the need to revise follow-up schedules to reduce the anxiety, costs, and radiation exposure of currently recommended follow-up strategy.

## Key Points

**Question:**

What are the outcomes of a guideline-based follow-up in patients affected by a gastrointestinal stromal tumor (GIST) at low-risk of recurrence?

**Findings:**

In this cohort study, 737 patients who underwent surgical resection for low-risk GIST treated at Italian Sarcoma Group referral centers were included and, with a median follow-up of 69.2 months, 42 patients (5.7%) experienced relapse; according to current guideline recommendations, this translates into 1 relapse detected every 170 computed tomography scans performed. Second tumors affected 25% of patients, representing the leading cause of death in this population.

**Meaning:**

The findings of this study suggest that, in low-risk GISTs, relapses are uncommon but observed even after more than 10 years; current follow-up schedules for low-risk GIST may need to be revised.

## Teaser

This cohort study examines the outcomes observed after guideline-based 5- and 10-year follow-up in patients with gastrointestinal stromal tumor at low risk of recurrence following surgery.

## Introduction

Gastrointestinal stromal tumor (GIST) is the most common mesenchymal tumor of the gastrointestinal tract with an expected incidence of approximately 10 to 15 cases per million population per year.^1^ The clinical behavior of GISTs shows wide variability, spanning from very indolent and slow-growing diseases to highly aggressive and metastatic ones. Through the years, several risk stratification models have been developed aiming to predict GIST behavior to guide the clinical decision-making process for localized disease.^2,3,4,5,6^ Within this frame, defining what is a true low-risk GIST is still debated. Despite small differences in setting the threshold regarding size and mitotic index, there is general agreement that smaller GISTs with low mitotic count are those with a lower risk of relapse.^2,5,7,8^ Beyond these minor differences, whatever tool is used, risk assessment has a paramount importance in the whole treatment strategy. Not only does it define patients who are candidates for (or should be evaluated for) adjuvant treatment with imatinib, it also suggests how to manage clinical surveillance after complete surgery.^9,10,11^ The intensity and timing of follow-up remains another matter of debate since little and weak evidence is currently available.^10,12,13^

As a general rule, follow-up is considered worthy if early recognition of relapse and its subsequent association with the disease course and outcome counterbalances the increased costs, radiation exposure, and medicalization of patients.^14^ Finding the correct balance is particularly challenging when the a priori probability of relapse is low. This greatly increases the number of examinations and tests needed to find a single relapse event, reducing the chances to detect a benefit in the few patients who experience disease recurrence. In GISTs, some evidence suggests the potential utility of routine follow-up, and European Society of Medical Oncology (ESMO) guidelines indicate routine monitoring of patients who undergo complete resection of their disease with intensity modulated according to risk stratification.^10^

In a previous retrospective work, the Italian Sarcoma Group found that earlier recognition of relapses might impact survival.^15^ Nonetheless, in the same series, the researchers were unfavorably impressed by the high number of computed tomographic (CT) scans (approximately 150) performed to detect 1 recurrence in the low-risk population. These data prompted us to assess in a larger cohort of low-risk patients whether high-intensity clinical surveillance is worth the effort and cost or, vice versa, we should revise our follow-up strategy.

## Methods

### Patients

From 18 institutional prospectively collected databases of Italian Sarcoma Group centers, we identified patients affected by GIST undergoing radical surgery between January 2001 and June 2019. We included only patients classified as having low risk of recurrence according to the Armed Forces Institute of Pathology criteria.^2^ Therefore, patients with tumor rupture at the time of surgery and/or treated with neoadjuvant or adjuvant imatinib were excluded. Our data set included patients with histologic diagnosis of GIST for whom the following clinical information was available: site of origin, tumor size (GISTs <2 cm of gastric origin were excluded given their potentially indolent behavior), mitotic index, microscopic surgical margins (R2 resections were excluded), and at least 2 years of follow-up and observation for patients who did not experience recurrence or death. We also collected other clinically relevant information (eg, mutational status, presence of tumor-related symptoms at diagnosis, such as bleeding or pain) that were not mandatory for inclusion in the present analysis. In case of recurrence, postrecurrence treatment information was collected whenever available. The study was conducted in accordance with the Declaration of Helsinki.^16^ Ethics committees and/or institutional review boards of the participating centers approved the study. Informed consent was obtained in a written form for study participants whenever applicable. For patients who died or were lost to follow-up, data collection was allowed by ethics committees and institutional review boards. We followed the Strengthening the Reporting of Observational Studies in Epidemiology (STROBE) reporting guideline for reporting our data.^17^

### Outcome of Interest

The primary end point of the study was the number of tests needed to identify a relapse according to ESMO guidelines follow-up plan.^10^ Secondary end points included relapse rate, relapse timing, disease-free survival (DFS), overall survival (OS), GIST-specific survival (GIST-SS), postrelapse OS (PR-OS), secondary tumor rate, and theoretical ionizing radiation exposure (assuming 8 mSv per each CT scan). In addition, we aimed to propose a new follow-up schedule for patients with low-risk GIST according to the observed results.

Patients were monitored according to ESMO guidelines follow-up schedule based on risk stratification.^10^ Thus, for patients with low- and very low-risk GIST, clinical examinations and CT scans of the abdomen and pelvis were performed every 6 months for 5 years, and then annually for up to 10 years. The diagnosis of non-GIST malignant tumors during follow-up was carefully recorded.

### Statistical Analysis

Statistical analysis was performed from December 15, 2022, to March 20, 2023, with SPSS, version 28.0 (IBM Corp) and R Jamovi, version 2.3.26.0 (R Foundation for Statistical Computing). Descriptive statistics for the following variables were analyzed: sex, age at diagnosis, tumor site, tumor size, mitotic index, mutational status, and symptoms at diagnosis. The χ^2^ and Fisher exact tests and/or the Mantel-Haenszel odds ratio (OR) estimates, where indicated, were used to compare qualitative variables. All survival end points (DFS, OS, GIST-SS, PR-OS) were estimated according to the Kaplan-Meier method.^18^ Disease-free survival was calculated from the date of surgery to the date of recurrence or death, whichever occurred first. For patients who had a diagnosis of a second tumor during follow-up, GIST DFS was censored at the date of the second tumor diagnosis unless it was radically treated and/or the 2 diseases could unambiguously be distinguished (eg, prostate-specific antigen–positive prostate cancer). However, GIST DFS was considered as an event of any biopsy or surgery showing that the identified deposit was consistent with GIST histologic characteristics (pathology report on metastasis).

Overall survival and GIST-SS were computed from the date of surgery to the date of death, and PR-OS was calculated from the date of first recurrence to the date of death. Patients who died from causes other than GIST were censored for GIST-SS but were considered as events for OS. Patients alive at the date of last follow-up were censored. Patients lost to follow-up were censored for the event of interest at the last date they were free from the event. In case of missing data (eg, symptoms at diagnosis, mutational status), all analyses were performed on the subgroup of patients for whom the information was available. Comparisons were performed using log-rank test and hazard ratio (HR) estimates calculated by Cox proportional hazards regression.^19^ We checked the proportional hazards assumption by visual inspection of the log-minus-log plots. When indicated, tests were 2-sided, and results are reported with 95% or IQRs. A *P* value ≤.05 was considered statistically significant.

## Results

**Figure 1.**
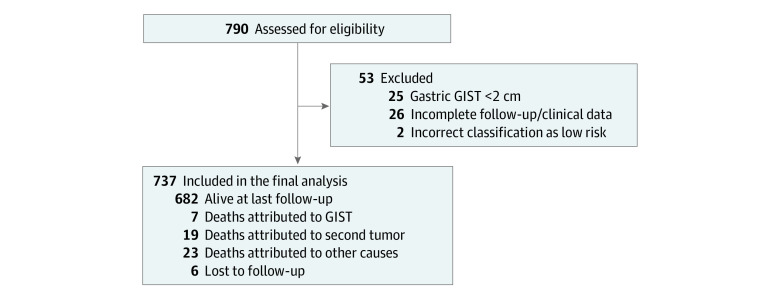
Patient Flowchart GIST indicates gastrointestinal stromal tumor.

A total of 790 patients affected by low-risk GIST were retrospectively identified in our institutional databases. After data cleaning and revision, 53 patients (6.7%) were excluded because of gastric GISTs less than 2 cm (so-called micro-GIST, 25 patients), incomplete follow-up or inadequate clinical data (26 patients), or incorrect classification in the low-risk category (2 patients reclassified as intermediate risk according to Armed Forces Institute of Pathology criteria). A total of 737 patients were included in the present analysis (Figure 1).

Of these patients, 360 were women (48.8%), 377 were men (51.2%), and median age at diagnosis was 63 (range, 18-86) years. Self-reported race was White or Caucasian for all patients. Primary tumor site distribution was as expected in this population, with a predominance of gastric GIST (68.1%). The Table reports other primary patient characteristics.

**Table.** Patient Demographic Characteristics

Characteristic	No. (%)
Patients eligible for analyses	737 (100)
Age at diagnosis, y	
Median (range)	63 (18-86)
<65	402 (54.5)
≥65	335 (45.5)
Sex	
Male	377 (51.2)
Female	360 (48.8)
Race^a^	
White or Caucasian	737 (100)
Tumor site	
Stomach	502 (68.1)
Duodenum	68 (9.2)
Small bowel	143 (19.4)
Large bowel or rectum	16 (2.2)
Other	8 (1.1)
Tumor size, cm	
<5	576 (78.2)
>5-10^b^	161 (21.8)
Mitotic count (per 50 HPF)	
≤5	713 (96.7)
6-10	24 (3.3)
Symptoms at diagnosis	
No	362 (49.1)
Yes	281 (38.1)
Not reported	94 (12.8)
Bleeding at diagnosis	
No	520 (70.6)
Yes	182 (24.7)
Not reported	35 (4.7)
Type of surgery	
Laparoscopic	214 (29.0)
Laparotomic	435 (59.0)
Endoscopic	48 (6.5)
Not reported	40 (5.4)
Radicality of surgery	
R0	699 (94.8)
R1	38 (5.2)
Second tumors	
Overall	187 (25.4)
Before GIST	80 (10.8)
Synchronous with GIST	51 (6.9)
After GIST	56 (7.6)
Mutations^c^	
Available mutational data	294 (39.9)
*KIT* ex11	102 (34.7)
*KIT* ex11del	53 (18.0)
*KIT* ex9	22 (7.5)
*KIT* ex13	8 (2.7)
*KIT* ex17	3 (1.0)
*PDGFRA* D842V	61 (20.7)
*PDGFRA* non-D842V	28 (9.5)
Other	17 (5.8)

Abbreviations: del, deletion; ex, exon; HPF, high-power field; *PDGFRA*, platelet-derived growth factor α.

^a^ Self-reported race was White or Caucasian for all patients.

^b^ Only gastric GISTs with mitoses less than 5 per 50 HPF.

^c^ Percentages of the different mutations are reported considering the available mutational data (294 cases).

To exclude biases related to geographic patient distribution, we randomly divided the patient population into 2 groups according to the different centers. We performed this random assignment twice. In both analyses, the 2 groups of patients were superimposable with no significant differences for all parameters in terms of outcomes and baseline characteristics. After this first check, we considered the whole population for all the subsequent analyses.

**Figure 2.**
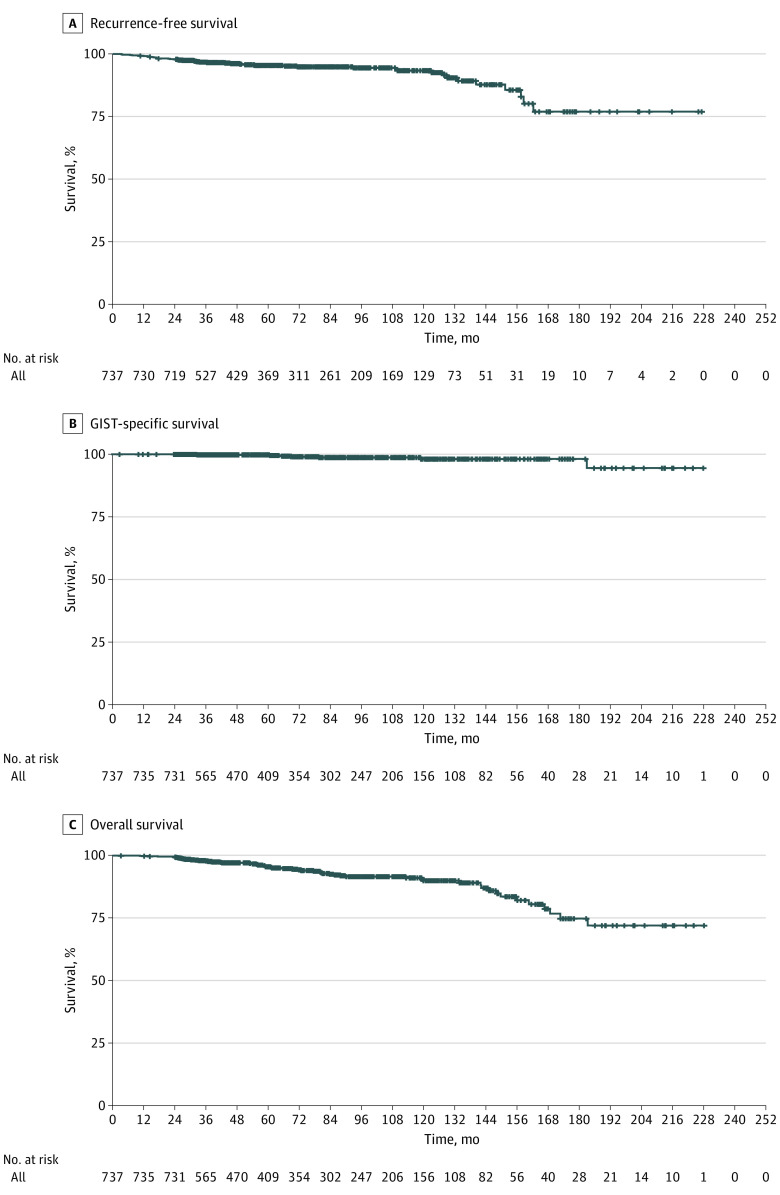
Survival Outcomes A, Recurrence-free survival. B, Gastrointestinal stromal tumor (GIST)–specific survival. C, Overall survival.

With a median follow-up of 69.2 (95% CI, 62.9-75.6) months, disease relapse occurred in 42 of 737 eligible patients (5.7%). Estimated survival rates at 5 years were 95.5% for DFS, 99.8% for GIST-SS, and 96.1% for OS, and 10-year survival rates were 93.4% for DFS, 98.1% for GIST-SS, and 91.0% for OS (Figure 2).

Of the 42 patients who experienced relapse, 9 (21%) had a local relapse (esophagus, n = 1; stomach, n = 5; duodenum, n = 1; small bowel, n = 2), 31 (74%) had distant metastases, and 2 (5%) had both local and distant relapses. Of the 33 patients who developed metastases, 15 (45.4%) involved the liver, 9 (27.3%) the peritoneum, 7 (21.2%) both liver and peritoneum, and 2 (6.1%) other sites (bone, spleen). These relapses were detected mainly in the first 2 years after primary surgery (15 of 42 [36%]), but we observed 9 of 42 relapses (21%) occurring after 10 years or more (eFigure 1 in Supplement 1).

Relapses were detected by CT scan in most of the cases (35 of 42 [83%]), while abdominal ultrasonography was able to detect a recurrence in 8 cases (19%) and endoscopy in 6 cases (14%). According to ESMO guidelines, these patients should have undergone a total of 7127 CT scans during their follow-up period, with a median of 10 (IQR, 4-14) CT scans per patient and a mean (SD) ratio of CT scans performed or expected of 0.83 (0.30). This finding indicates that we detected 1 recurrence for about every 170 CT scans performed, which translates into an approximate 0.6% probability to detect a recurrence with a CT scan. In terms of radiation exposure, this translates into a median exposure of 80 (IQR, 32-112) mSv per patient.

### Relapse According to Primary Site

**Figure 3.**
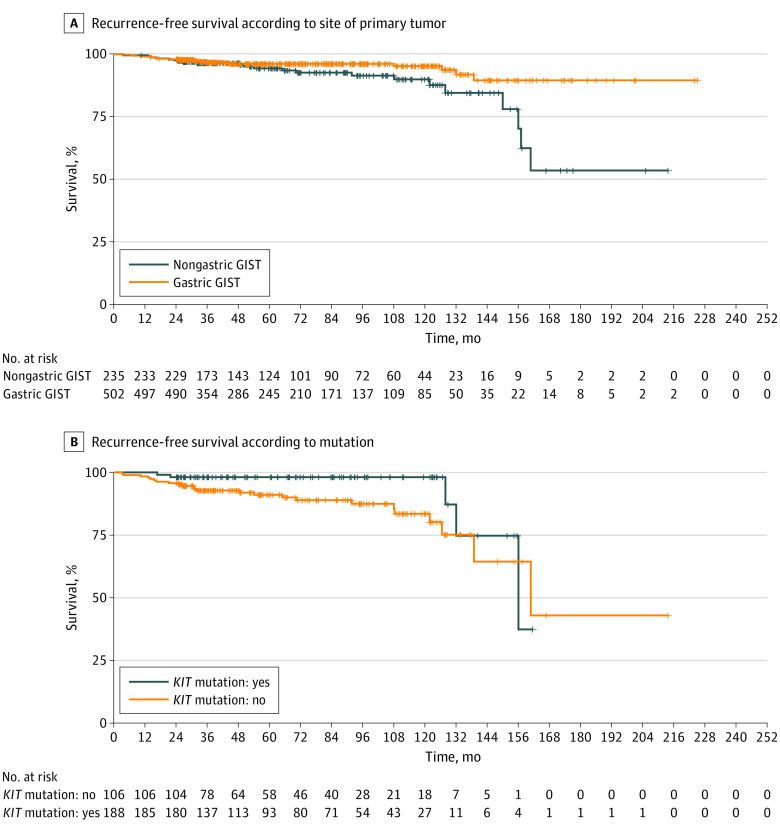
Recurrence-Free Survival According to Site and Mutational Analysis A, Recurrence-free survival according to site of primary tumor. B, Recurrence-free survival according to *KIT* vs other mutations. GIST indicates gastrointestinal stromal tumor.

Nongastric primary was associated with a higher relapse risk compared with gastric ones (log-rank *P* = .01; HR, 2.09; 95% CI, 1.14-3.83; *P* = .02), with an estimated DFS for nongastric vs gastric primary of 94.3% vs 96.1% at 5 years and 89.9% vs 95.3% at 10 years (Figure 3A).

Patients with tumor-related symptoms at the time of diagnosis showed a nonsignificant risk of relapse (log-rank *P* = .06; HR, 1.85; 95% CI, 0.96-3.56; *P* = .07). This finding seems to be mainly associated with gastric GISTs where the presence of symptoms at diagnosis was associated with a higher relapse rate (6.7% vs 2.4%; OR, 2.90; 95% CI, 1.07-7.87; *P* = .04), while it was not observed in nongastric tumors (9.8% vs 8.0%; OR, 1.26; 95% CI, 0.49-3.23; *P* = .64).

### Relapse According to Mutational Analysis

Among the 294 patients (39.9%) for whom mutational analysis was available, patients affected by GISTs with mutations in *KIT* had a significantly higher risk of relapse (log-rank *P* = .03) compared with patients with molecular alterations involving *PDGFRA* (platelet-derived growth factor α) or other genes (eg, *BRAF*, *SDH*, [succinate dehydrogenase] *NF-1* [(neurofibromatosis type 1]) (HR, 2.77; 95% CI, 1.05-7.27; *P* = .04). In patients with mutations in *KIT* vs other genes, the estimated DFS was 91.1% vs 98.1% at 5 years and 83.5% vs 98.1% at 10 years (Figure 3B).

### Treatment of Relapse

All patients received imatinib at the time of relapse. After or during imatinib treatment, 17 patients (40%) underwent surgery for relapsed disease. Of these patients, 4 had local relapse, 12 had metastatic disease, and 1 had both local and metastatic disease. Patients who received surgery for relapsed disease experienced a significantly better PR-OS compared with patients who did not (median PR-OS at 5 years was not reached [NR]; 95% CI, NR-NR, and 100%; 95% CI, 100%-100% for patients who underwent surgery for relapse vs 169.9 months; 95% CI, 49.4%-NR, and 58.2%; 95% CI, 36.8%-91.9% for patients who did not; log-rank *P* = .005), and no patients died in this group at the time of data lock (eFigure 2 in Supplement 1).

### Second Tumors

Second malignant tumors were a common event in this patient population, being observed in 187 of 737 patients (25%). Of these second tumors, 80 were diagnosed before GIST, 51 at the same time of GIST, and 56 during follow-up. Of the 56 second tumors diagnosed during follow-up, 28 were incidentally detected during follow-up examinations and visits. Deaths attributed to second tumors greatly exceed the number of deaths attributed to GIST (19 vs 7 events) and represented the leading cause of death in this population (39% of all events).

### Proposal of Follow-Up Schedule Revision

**Figure 4.**
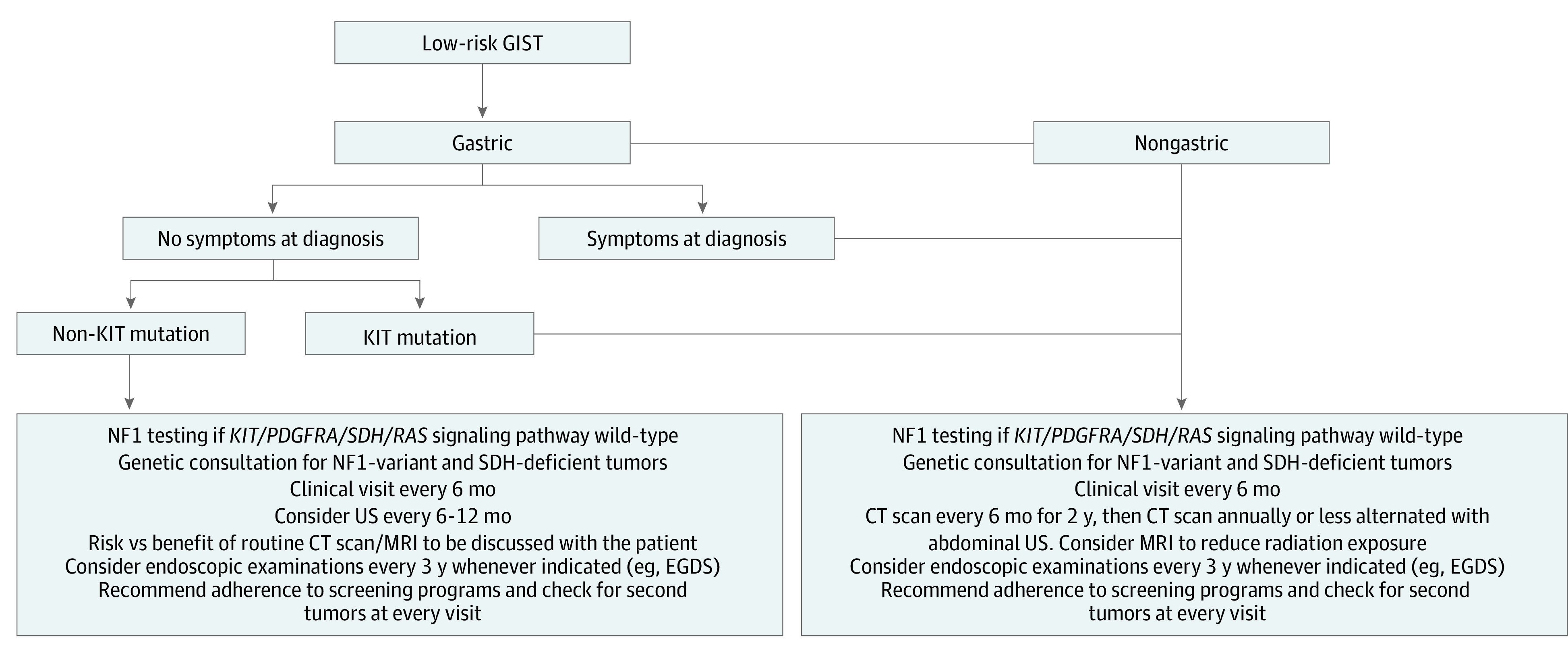
Revised Surveillance Algorithm CT indicates computed tomography; EGDS, esophagogastroduodenoscopy; GIST, gastrointestinal stromal tumor; MRI, magnetic resonance imaging; NF1, neurofibromatosis 1; and US, ultrasonography.

As suggested by our results, follow-up might be revised according to site, mutational analysis, and symptoms at diagnosis. We recommend closer surveillance for nongastric GISTs and gastric GISTs with symptoms at diagnosis or bearing *KIT* mutation. In this subset of patients, we suggest performing a CT scan twice a year in the first 2 years of follow-up and annually thereafter. However, in asymptomatic patients affected by low-risk gastric GISTs, the true benefit of surveillance is left to be demonstrated. Accordingly, yearly abdominal magnetic resonance imaging or CT scan, or even ultrasonography, seem to be a reasonable option. Figure 4 shows a proposal of a new algorithm for surveillance. Considering the associated risk, confirmed in several series,^20,21,22^ a nonnegligible benefit of surveillance might be an earlier detection of second tumors.

## Discussion

To our knowledge, our study represents the largest series to date evaluating the role of follow-up after radical surgery in patients with GIST classified at low-risk of relapse according to Armed Forces Institute of Pathology criteria. With a median follow-up of nearly 6 years, we observed that the risk of relapse in this patient population remains low with an overall relapse rate of 5.7% (21% local, 74% distant, 5% both).^5^ However, our data suggest that any GIST entails a malignant potential. Notably, about one-fifth of the relapses in our series were detected after more than 10 years from primary surgery. Moreover, the observed risk of having a second malignant tumor was observed in up to 1 of every 4 patients (25%).

Given the overall very good prognosis with a 10-year GIST-SS above 95% and the potential need to extend follow-up beyond 10 years, these data highlight the need of a shared decision-making process with patients regarding follow-up timing and procedures. On one side we should consider the potential burden of follow-up in terms of costs (1 relapse detected in approximately every 170 CT scans performed), ionizing radiation exposure with a mean of 8 to 10 mSv per CT scan (providing a lifetime additional risk of fatal cancer per each examination of approximately 1 in 2000),^23,24^ and increased medicalization and anxiety of patients^25,26,27^ that in most patients could be considered resolved by surgery alone. On the other side, a scheduled follow-up might allow an earlier recurrence detection with a lower tumor burden and/or before symptoms occurrence and might increase the sense of control for some patients.^15,26^ This might affect subsequent treatments and increase the chance of starting medical therapy with the lowest tumor burden possible, which is the most important determinant of long-term disease control after recurrence.^14,28^ Surgery might be an option in patients with relapse,^10,15,29,30,31,32,33,34^ and, in our series we did not observe any death event after complete tumor removal. However, it is left to be demonstrated whether it is only a matter of selection bias. In the absence of robust prospective data, surgery in the metastatic or relapsed setting might be proposed when complete resection can be achieved at a reasonable price for the patient.^10^

In this population of low-risk GISTs, patients with *KIT*-mutant tumors experienced a worse DFS compared with those who had GISTs harboring non-*KIT* molecular alterations. This is in line with previous data not restricted for risk stratification that showed a worse DFS for *KIT*-mutant vs *PDGFRA*-mutant GISTs. In that work, mutational analysis, gastric origin, and tumor size significantly correlated with DFS.^13^ Consistently, also in our series, gastric origin was associated with a lower relapse risk, while the restriction to the low-risk population did not allow us to detect a significant impact of tumor size in terms of RFS. Within gastric GISTs, we found a nonsignificant increased risk of relapse for patients who had tumor-related symptoms at diagnosis (eg, bleeding).

In our data set, mutational analysis was available for 39.9% of patients. This is somewhat expected considering that some centers did not routinely perform mutational analysis for low-risk GISTs, given the absence of therapeutic implications. For the same reason, it was not always possible to compare mutational analysis at recurrence with the one performed on the primary tumor, especially for late recurrences. This might open the unsolved issue of whether local recurrences occurring after more than 10 years should be considered true relapses or second GISTs. That said, the possibility to detect GIST recurrences has been reported even after more than 15 years in another series.^20^ When mutational analysis of the primary tumor was available and/or tumor tissue samples were of adequate quality, we repeated mutational analysis and found the same molecular alteration detected at diagnosis also in 5 GISTs relapsing after more than 10 years.

### Limitations

Our study has limitations, mainly related to its retrospective nature. In particular, in our data set, rectal GISTs were quite underrepresented compared with other series.^20,35^ This is probably due to the exclusion of patients who were treated with neoadjuvant imatinib, which is rather common for rectal GISTs compared with other sites, in the attempt to preserve sphincter function.^35,36^

Another potential limitation is that the retrospective evaluation of relapses in low-risk GISTs from Italian Sarcoma Group referral centers might slightly overestimate their incidence due to the loss of low-risk patients who did not experience relapse and were treated outside these centers. Although we did our best to collect data on the whole population also from peripheral centers referring patients at the time of relapse, we cannot exclude that relapse incidence remains slightly inflated. Nonetheless, in case of relapse rate inflation, the true ratio of relapses detected per CT scans performed might be even lower than the 1 in 170 observed in our data set, further supporting the need to revise the follow-up for this patient population.

With these potential limitations, since no prospective trial on this topic can be foreseen soon, or maybe never, our study depicts a picture of the clinical setting in the management of low-risk GISTs with data coming from main Italian Sarcoma Group referral centers that might help in the shared decision-making process with patients regarding follow-up strategy. Taken together, our data suggest that the follow-up for patients with low-risk GIST be less intensive, particularly for gastric tumors without symptoms at diagnosis. We suggest reducing the burden of unnecessary ionizing radiation exposure and costs by lowering the number of CT scans and increasing the use of other imaging strategies. Two-thirds of the relapses reported herein involved the liver, and these can be routinely evaluated by means of ultrasonography. Magnetic resonance imaging also represents an alternative option; however, there are well-known limitations related to costs, timing of the examinations, and patients’ potential refusal due to claustrophobia (incidence up to 10%-20% according to available literature).^37^ When considering costs, the new algorithm proposed for surveillance requires that mutational analysis be performed. Although it is also often performed in low-risk GISTs in many reference sarcoma centers, the potential additional financial burden of mutational analysis for these patients can be counterbalanced by the reduction of radiologic examinations and ionizing radiation exposure.

When considering how to manage follow-up intensity in low-risk GISTs, the burden of second tumors must be considered. In this study population, second tumors were detected more frequently than GIST relapses during follow-up and our data are consistent with several other series.^20,21,22^ Therefore, patients affected by low-risk GIST should be encouraged to adhere to screening programs and checked for signs of second tumors at the time of each follow-up visit.

## Conclusions

In this retrospective cohort study, we report what is, to our knowledge, the largest series to date on low-risk GIST surveillance. According to the observed results, we suggest revising the follow-up currently suggested by ESMO guidelines by reducing the number of CT scans to be performed. Furthermore, recurrences were identified after 10 years of follow-up, requiring awareness about very late relapses. We also observed that second tumors were a relatively common event in patients with low-risk GISTs and represented the leading cause of death.

## Notes

Supplement 1. **eFigure 1.** Distribution of Relapses Through the Years of Follow-Up **eFigure 2.** Post-Recurrence Overall Survival According to Surgical Resection of Relapsed Disease

Supplement 2.
Nonauthor Collaborators of the Italian Sarcoma Group

Supplement 3.
Data Sharing Statement
