# Error in Affiliation

**DOI:** 10.1001/jamanetworkopen.2023.54891

**Published:** 2024-01-17

In the Original Investigation titled “Guideline-Based Follow-Up Outcomes in Patients With Gastrointestinal Stromal Tumor With Low Risk of Recurrence: A Report From the Italian Sarcoma Group,”^1^ published November 6, 2023, there was an error in an author affiliation. The affiliation for Silvia Stacchiotti, MD, given as “San Luigi Gonzaga University Hospital, Orbassano, Italy” should have been “Medical Oncology Unit 2, Medical Oncology Department, Fondazione IRCCS Istituto Nazionale dei Tumori di Milano, Milan, Italy.” This article has been corrected.^1^
