# Supplementary material for: Guideline-Based Follow-Up Outcomes in Patients With Gastrointestinal Stromal Tumor With Low Risk of Recurrence: A Report From the Italian Sarcoma Group
Source: JAMA Netw Open. 2023 Nov 6;6(11):e2341522. doi: 10.1001/jamanetworkopen.2023.41522 (PMC10628737; doi:10.1001/jamanetworkopen.2023.41522)
Supplement: Supplement 1. — eFigure 1. Distribution of Relapses Through the Years of Follow-Up eFigure 2. Post-Recurrence Overall Survival According to Surgical Resection of Relapsed Disease [file jamanetwopen-e2341522-s001.pdf]

Supplementary Online Content

D'Ambrosio L, Fumagalli E, De Pas TM, et al. Guideline-based follow-up outcomes in patients with gastrointestinal stromal tumor with low risk of recurrence. *JAMA Netw Open*. 2023;6(11):e2341522. doi:10.1001/jamanetworkopen.2023.41522

eFigure 1. Distribution of Relapses Through the Years of Follow-Up

eFigure 2. Post-Recurrence Overall Survival According to Surgical Resection of Relapsed Disease

This supplementary material has been provided by the authors to give readers additional information about their work.

© 2023 D'Ambrosio L et al. *JAMA Network Open.*

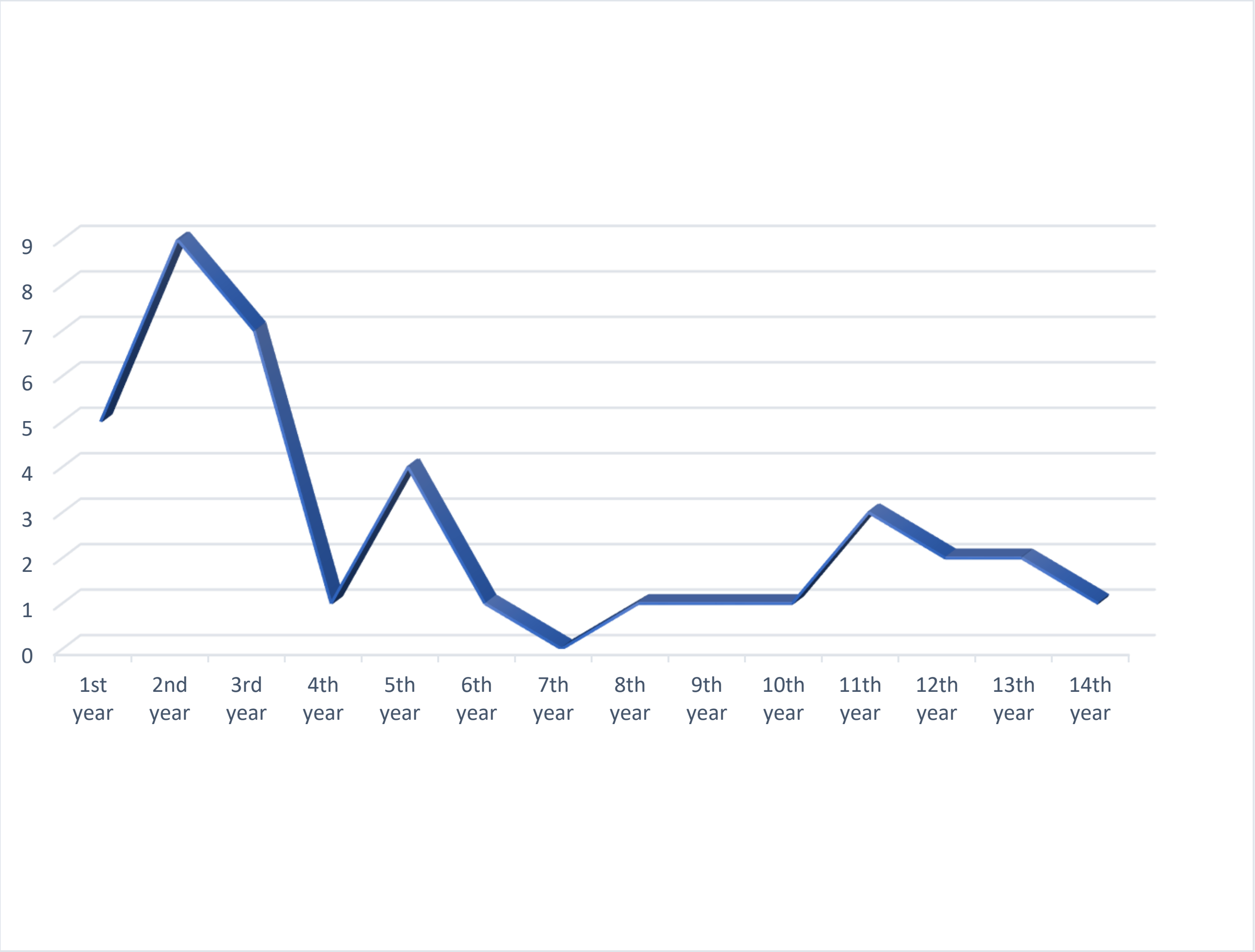

eFigure 1. Distribution of relapses through the years of follow-up.

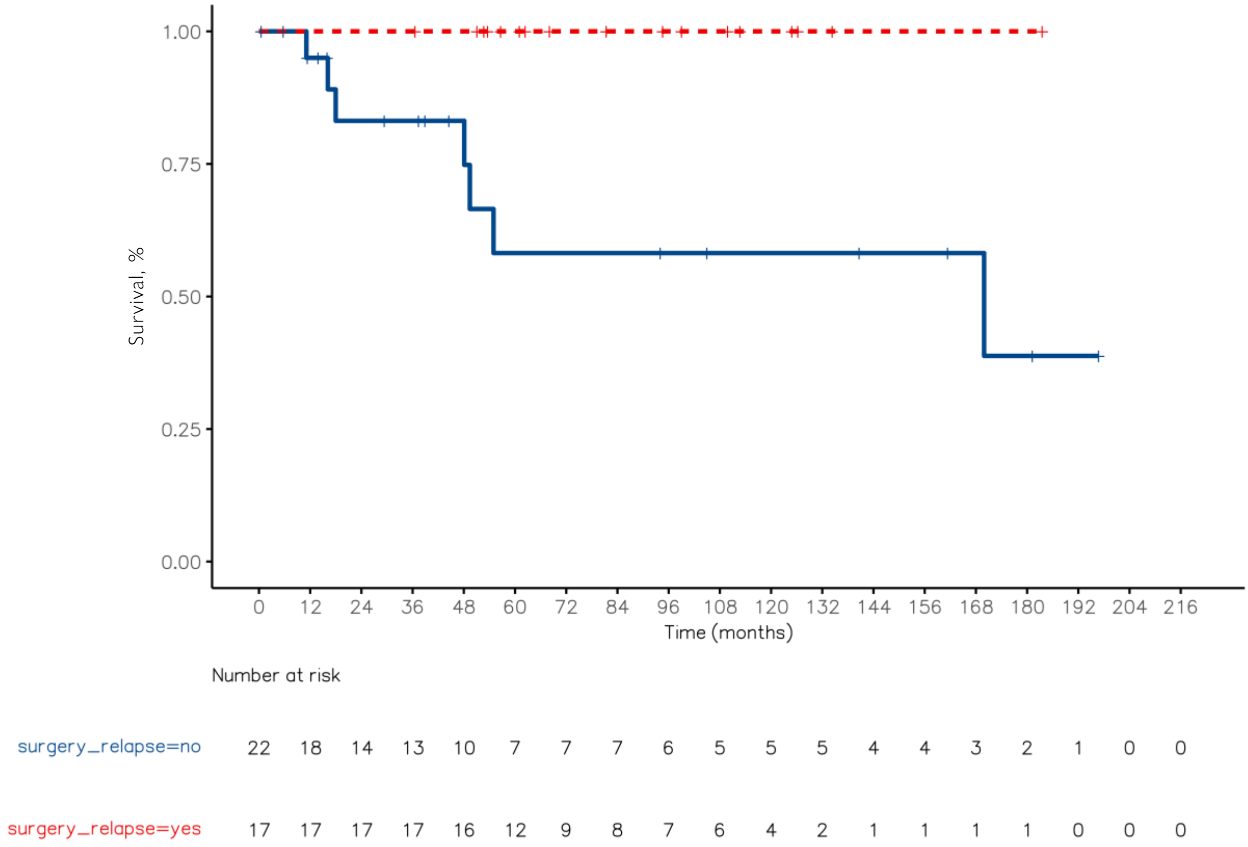

eFigure 2. Post-recurrence overall survival according to surgical resection of relapsed disease. Red line surgery, blue line no surgery.
