# Supplementary material for: Guideline-Based Follow-Up Outcomes in Patients With Gastrointestinal Stromal Tumor With Low Risk of Recurrence: A Report From the Italian Sarcoma Group
Source: JAMA Netw Open. 2023 Nov 6;6(11):e2341522. doi: 10.1001/jamanetworkopen.2023.41522 (PMC10628737; doi:10.1001/jamanetworkopen.2023.41522)
Supplement: Supplement 2. — Nonauthor Collaborators of the Italian Sarcoma Group [file jamanetwopen-e2341522-s002.pdf]

Supplemental Online Content: Nonauthor Collaborators
*First name, last name, and suffix (if applicable) are required and will appear in PubMed.

***Group Name(s): Italian Sarcoma Group**

| ***First Name and Middle Initial(s)** | ***Last Name** | ***Suffix (eg, Jr, III)** | Academic Degrees | Institution | Location (city, state/province, country) | Role or Contribution, eg, chair, principal investigator | Group (if more than 1 Group listed in the byline) and/or Subgroup (eg, Steering Committee) |
|---|---|---|---|---|---|---|---|
| Gianluca | Ignazzi | | | Italian Sarcoma Group | | ISG Trial Center - coordinator | Italian Sarcoma Group |
| Viviana | Apolloni | | | Italian Sarcoma Group | | ISG Trial Center - study coordinator | Italian Sarcoma Group |
| Laura | Abate Daga | | | Italian Sarcoma Group | | ISG administrative office | Italian Sarcoma Group |
| Giuseppe | Bianchi | | | Italian Sarcoma Group | | ISG administrative office | Italian Sarcoma Group |
