# Supplementary material for: Guideline-Based Follow-Up Outcomes in Patients With Gastrointestinal Stromal Tumor With Low Risk of Recurrence: A Report From the Italian Sarcoma Group
Source: JAMA Netw Open. 2023 Nov 6;6(11):e2341522. doi: 10.1001/jamanetworkopen.2023.41522 (PMC10628737; doi:10.1001/jamanetworkopen.2023.41522)
Supplement: Supplement 3. — Data Sharing Statement [file jamanetwopen-e2341522-s003.pdf]

Data Sharing Statement

D'Ambrosio. Guideline-Based Follow-Up Outcomes in Patients With Gastrointestinal Stromal Tumor With Low Risk of Recurrence. *JAMA Netw Open*. Published November 06, 2023. doi:10.1001/jamanetworkopen.2023.41522

Data
Data available: Yes
Data types: Other (please specify)
Additional Information: Deidentified participant data might be evaluated for sharing only after evaluation and approval from Ethic Committee and Data Protection Officer evaluation.
How to access data: Deidentified participant data might be evaluated for sharing only after evaluation and approval from Ethic Committee and Data Protection Officer evaluation.
When available: With publication

Supporting Documents
Document types: Other (please specify)
Additional Information: Deidentified participant data might be evaluated for sharing only after evaluation and approval from Ethic Committee and Data Protection Officer evaluation.
How to access documents: Deidentified participant data might be evaluated for sharing only after evaluation and approval from Ethic Committee and Data Protection Officer evaluation.
When available: With publication

Additional Information
Who can access the data: Deidentified participant data might be evaluated for sharing only after evaluation and approval from Ethic Committee and Data Protection Officer evaluation.
Types of analyses: Deidentified participant data might be evaluated for sharing only after evaluation and approval from Ethic Committee and Data Protection Officer evaluation.
Mechanisms of data availability: Deidentified participant data might be evaluated for sharing only after evaluation and approval from Ethic Committee and Data Protection Officer evaluation. Investigator support and involvement in data analysis will be required.
